# Predicting cerebral infarction in COPD patients: an individualized nomogram based on arterial oxygen saturation

**DOI:** 10.3389/fmed.2025.1675147

**Published:** 2025-12-18

**Authors:** Deyi Zhou, Xiaomi Chen, Lijuan Zeng, Boyang Xiao, Yuli Cai, Huimin Chen, Zhaojun Chen, Qinghua Chen, Jingjing Pan, Feiju Chen, Sihan Lin, Xing Li, Xinyao Liu, Junfen Cheng, Weimin Yao, Riken Chen, Guangbin Liang

**Affiliations:** 1 The Second Affiliated Hospital of Guangdong Medical University, Zhanjiang, Guangdong Province, China; 2 Guangdong Pharmaceutical University, Guangzhou, Guangdong Province, China; 3 Affiliated Hospital of Guangdong Medical University, Zhanjiang, Guangdong Province, China

**Keywords:** chronic obstructive pulmonary disease, cerebral infarction, arterial oxygen saturation, nomogram, hypoxia

## Abstract

**Objective:**

To investigate the correlation between arterial oxygen saturation (SaO_2_) and the occurrence of cerebral infarction (CI) in patients with chronic obstructive pulmonary disease (COPD), and to develop a nomogram model based on SaO_2_ to predict the probability of CI in COPD patients.

**Methods:**

This retrospective study analyzed the clinical data of 846 COPD patients admitted to the Affiliated Hospital of Guangdong Medical University from June 2018 to December 2019. Logistic regression analysis was used to identify risk factors for CI, and the Wald chi-square test was applied to select predictors for inclusion in the nomogram. The performance of the model was evaluated and internally validated. External validation was conducted using data from 290 COPD patients prospectively enrolled at the Second Affiliated Hospital of Guangdong Medical University between January and October 2024.

**Results:**

A total of 846 COPD patients were included, with 592 assigned to the training cohort and 254 to the internal validation cohort. Predictive factors incorporated into the nomogram included systemic immune-inflammation index (SII), age, hypertension, cardiovascular disease (CVD), paraplegia, and SaO_2_. The nomogram demonstrated strong predictive accuracy and calibration, with AUCs of 0.85 (95% CI: 0.82–0.89) in the training cohort, 0.89 (95% CI: 0.85–0.94) in the internal validation cohort, and 0.90 (95% CI: 0.86–0.94) in the external validation cohort.

**Conclusion:**

COPD is prone to cerebral infarction, and we verified the relationship between COPD and cerebral infarction through a visualogram model. The incidence of cerebral infarction in COPD patients is affected by systemic immune-inflammatory index (SII), age, hypertension, cardiovascular disease (C), hemiplegia, and SaO_2_, and the nomogram model for risk prediction based on SaO_2_ has good predictive efficacy, which can provide a reference forbral infarction in COPD patients in clinical practice. However, this study was retrospective, the sample size of the different subtypes of ischemic stroke was small, the risk analysis of each subtype of ischemic stroke could not be performed, and there may be some unmeasured confounding factors, which had a certain impact on the of the study. In the future, we need multi-center prospective studies to verify the effectiveness and practicality of the nomogram, and the basic mechanism of each risk factor also to be studied.

## 1 Background

Chronic obstructive pulmonary disease (COPD) and stroke are among the leading causes of morbidity and mortality worldwide (1, 2). Chronic obstructive pulmonary disease (COPD) is a chronic inflammatory airway disease characterized by airflow limitation. It predominantly affects middle-aged and elderly individuals and is often associated with a long history of smoking. COPD not only impairs the structure and function of the respiratory system but also causes varying degrees of damage to other organs or systems. This severely compromises patients' quality of life and places a substantial economic burden on families and society (3).

In recent years, domestic and foreign scholars (4) have reported that COPD, including pulmonary heart disease, has a thrombotic state Thrombotic state refers to the pathophysiological changes that occur in blood cellular and (or) non-cellular components that are prone to thrombosis (5), which can lead to the occurrence of thrombotic diseases, mainly pulmonary embolism, limb venous thrombosis, and CI. The main mechanisms its occurrence are divided into the following two points: (1) Chronic persistent hypoxia: COPD can lead to a state of chronic persistent hypoxia in the body which is prone to respiratory failure, causing hypoxemia, carbon dioxide retention, secondary polycythemia, increased blood viscosity, increased fibrinogen, increased D-dimer hypercoagulable state of blood, vascular wall damage, etc., which are all secondary risk factors for CI (6). (2) Systemic inflammatory response:COPD is accompanied by significant systemic inflammatory responses, marked by elevated levels of acute-phase proteins (e.g., C-reactive protein [CRP], fibrinogen), increased white blood cell and band cell counts due to bone marrow stimulation, and elevated cytokines such as interleukin-1 (IL-1), tumor necrosis factor-alpha (TNF-α), and IL-6, all of which may activate the vascular endothelium (7). Some of these circulating biomarkers are known to be associated with the severity of atherosclerosis, a major contributor to CI (8).

COPD complicated by CI is not uncommon. CI is characterized by ischemic necrosis or softening of brain tissue due to cerebral hypoxia or ischemia (9). When CI coexists with COPD, its clinical features are often atypical, including symptoms such as fever, cough, sputum production, dyspnea, dizziness, headache, slow responses, drowsiness, coma, aphasia, and varying degrees of limb paralysis, making it easily misdiagnosed (10, 11). Although previous studies have reported that COPD is prone to cerebral infarction complications (12–14), its related risk factors have not been further. Therefore, identifying risk factors for CI in COPD patients is of critical importance for timely prevention and treatment, aiding in disease management and improving patient outcomes. We constructed a column line graph model based on SaO_2_, and the application of this model can predict the probability of COPD patients with CI.

## 2 Materials and methods

### 2.1 General data

The training and internal validation cohorts consisted of 846 patients diagnosed with COPD who were admitted to the Affiliated Hospital of Guangdong Medical University between June 2018 and December 2019. Among these, 148 patients were diagnosed with cerebral infarction (CI) and 698 were not. Using RStudio, the dataset was randomly divided into a training cohort (*n* = 592) and an internal validation cohort (*n* = 254) in a 7:3 ratio. For external validation, data were prospectively collected from 290 COPD patients admitted to the Second Affiliated Hospital of Guangdong Medical University between January and October 2024. All participants met the diagnostic criteria for COPD (15), which is based on the presence of persistent airflow limitation confirmed by pulmonary function testing. The core criterion is a post-bronchodilator forced expiratory volume in 1 s to forced vital capacity ratio (FEV1/FVC) < 0.70, confirming persistent airflow limitation. Patients with incomplete or missing data were excluded.

This study was conducted in accordance with the Declaration of Helsinki and received approval from the Medical Ethics Committee of the Second Affiliated Hospital of Guangdong Medical University (PJKT2025-04-019) and the Affiliated Hospital of Guangdong Medical University (PJKT2025-099). Informed consent was obtained from all participants. For those unable to provide consent personally, informed consent was obtained from their legal guardians.

### 2.2 Data collection

The clinical data collected included general demographics (age, sex), comorbidities (diabetes, cardiovascular disease, hypertension, paraplegia, hyperlipidemia, and CI), and laboratory test results: arterial oxygen saturation (SaO_2_), prothrombin time, activated partial thromboplastin time, creatinine, monocytes, hematocrit, uric acid, urea, γ-glutamyl transferase, total bilirubin, alanine aminotransferase, aspartate aminotransferase, hemoglobin, and the systemic immune-inflammation index (SII). The diagnosis of CI was based on internationally recognized diagnostic criteria (16). The SII was calculated based on baseline peripheral blood tests, using the formula: (17):


$$\begin{array}{l} SII\;=\;Platelet\;count\;\times \;Neutrophil\;count/Lymphocyte\;count \end{array}$$

### 2.3 Statistical analysis

All statistical analyses were performed using RStudio, with a significance threshold set at *p* < 0.05. Continuous variables were expressed as mean ± standard deviation, while categorical variables were presented as frequencies and percentages. The chi-square test or Fisher's exact test was used to analyze categorical variables, and the *t*-test or Wilcoxon rank-sum test was applied to continuous variables.

Baseline characteristics in the training cohort were analyzed, and variables with *p* < 0.05 were included in the logistic regression analysis. The Wald chi-square test was employed to screen variables for inclusion in the nomogram model predicting CI occurrence among COPD patients. The discriminative ability of the model was assessed using the area under the receiver operating characteristic (ROC) curve (AUC). Calibration and clinical utility were evaluated through calibration curves and decision curve analysis (DCA), respectively.

## 3 Results

### 3.1 Comparison between training and internal validation cohorts

Distribution of cerebral infarction subtypes in the training cohort according to the CISS classification in Table 1. As shown in Table 2, a balance test was conducted between the training and internal validation cohorts. There were statistically significant differences in sex (*p* = 0.02) and hyperlipidemia (*p* = 0.031) between the two groups, while no statistically significant differences were observed for the remaining variables. Since sex and hyperlipidemia were not included in the nomogram model, these differences did not impact the final results.

**Table 1:** Distribution of cerebral infarction subtypes in the training cohort according to the CISS classification.

**CISS subtype**	***n* (%)**
Total	106 (100.00)
Large artery atherosclerosis (LAA)	36 (33.96)
Cardiogenic stroke (CS)	25 (23.58)
Penetrating artery disease (PAD)	22 (20.75)
Other etiology (OE)	7 (6.60)
Undetermined etiology (UE)	16 (15.10)

CISS, Chinese ischemic stroke subclassification.

Data are presented as number of patients (n) with percentage of the total cohort (%) in parentheses. Percentages may not sum to 100.00% due to rounding.

**Table 2:** Baseline characteristics comparison of the training and internal validation cohorts.

**Variables**	**Total (*n* = 846)**	**Test (*n* = 254)**	**Train (*n* = 592)**	**Statistic**	***p*-Value**
SII (10^9^/L)	1,536.90 ± 1,554.79	1,429.88 ± 1,287.35	1,582.82 ± 1,655.24	*t* = 1.721	0.19
Age (years)	75.78 ± 10.38	76.51 ± 9.87	75.47 ± 10.58	*t* = 1.794	0.181
SaO_2_ (%)	93.89 ± 4.29	94.07 ± 4.13	93.81 ± 4.36	*t* = 0.653	0.419
PT (s)	12.90 ± 2.54	13.09 ± 4.01	12.82 ± 1.53	*t* = 2.041	0.153
APTT (s)	33.42 ± 7.61	32.95 ± 7.41	33.62 ± 7.69	*t* = 1.391	0.239
Cr (μmol/L)	84.49 ± 64.00	83.14 ± 51.73	85.06 ± 68.62	*t* = 0.16	0.689
MON (10^9^/L)	0.70 ± 0.67	0.64 ± 0.30	0.73 ± 0.77	*t* = 2.879	0.09
HB (g/L)	123.74 ± 21.95	124.79 ± 25.03	123.29 ± 20.49	*t* = 0.831	0.362
HCT (%)	37.28 ± 9.91	37.86 ± 8.30	37.03 ± 10.52	*t* = 1.241	0.266
UR (mmol/L)	6.18 ± 3.72	6.15 ± 3.81	6.19 ± 3.69	*t* = 0.018	0.893
UA (μmol/L)	314.80 ± 111.70	324.75 ± 121.87	310.54 ± 106.87	*t* = 2.886	0.09
GGT (U/L)	41.90 ± 52.17	42.09 ± 47.87	41.82 ± 53.95	*t* = 0.005	0.945
TBIL (μmol/L)	11.39 ± 8.83	11.50 ± 7.40	11.34 ± 9.38	*t* = 0.056	0.814
ALT (U/L)	23.00 ± 57.39	21.37 ± 28.91	23.70 ± 65.95	*t* = 0.295	0.587
AST (U/L)	25.71 ± 32.66	26.79 ± 37.01	25.24 ± 30.63	*t* = 0.397	0.529
**Hypertension**				χ^2^ = 0.604	0.437
No	506 (59.81%)	157 (61.81%)	349 (58.95%)		
Yes	340 (40.19%)	97 (38.19%)	243 (41.05%)		
**CVD**				χ^2^ = 1.448	0.229
No	527 (62.29%)	166 (65.35%)	361 (60.98%)		
Yes	319 (37.71%)	88 (34.65%)	231 (39.02%)		
**Paraplegia**				χ^2^ = 0.323	0.57
No	748 (88.42%)	227 (89.37%)	521 (88.01%)		
Yes	98 (11.58%)	27 (10.63%)	71 (11.99%)		
**CI**				χ^2^ = 0.231	0.631
No	698 (82.51%)	212 (83.46%)	486 (82.09%)		
Yes	148 (17.49%)	42 (16.54%)	106 (17.91%)		
**Gender**				χ^2^ = 5.369	**0.02**
Female	215 (25.41%)	78 (30.71%)	137 (23.14%)		
Male	631 (74.59%)	176 (69.29%)	455 (76.86%)		
**DM**				χ^2^ = 1.108	0.292
No	730 (86.29%)	224 (88.19%)	506 (85.47%)		
Yes	116 (13.71%)	30 (11.81%)	86 (14.53%)		
**Hyperlipidemia**				χ^2^ = 4.652	**0.031**
No	745 (88.06%)	512 (86.49%)	233 (91.73%)		
Yes	101 (11.94%)	80 (13.51%)	21 (8.27%)		

SII, systemic immune-inflammation index; SaO_2_, arterial oxygen saturation; PT, prothrombin time; APTT, activated partial thromboplastin time; Cr, creatinine; MON, monocyte; HB, hemoglobin; ALT, alanine aminotransferase; AST, aspartate aminotransferase; CVD, cardiovascular disease; CI, cerebral infarction; DM, diabetes mellitus. Bold values indicate that the variable is statistically significant at *P* < 0.05.

Baseline characteristics of the training cohort are summarized in Table 3. A total of 486 non-CI patients and 106 CI patients were included. The mean age of the cohort was 75.47 ± 10.58 years. There were 455 males (76.86%) and 137 females (23.14%). Compared with the non-CI group, patients in the CI group tended to be older and had higher hematocrit, alanine aminotransferase (ALT), and systemic immune-inflammation index (SII) levels, along with lower arterial oxygen saturation (SaO_2_; Table 3).

**Table 3:** Baseline characteristics of the training cohort.

**Variables**	**Total (*n* = 592)**	**No cerebral infarction (*n* = 486)**	**Cerebral infarction (*n* = 106)**	**Statistic**	***p*-Value**
SII (10^9^/L)	1,582.82 ± 1,655.24	1,424.78 ± 1,256.13	2,307.39 ± 2,736.33	*t* = 25.78	**< 0.001**
Age (years)	75.47 ± 10.58	74.15 ± 10.58	81.51 ± 8.28	*t* = 45.19	**< 0.001**
SaO_2_ (%)	93.81 ± 4.36	94.22 ± 4.20	91.95 ± 4.63	*t* = 24.53	**< 0.001**
PT (s)	12.82 ± 1.53	12.83 ± 1.58	12.74 ± 1.28	*t* = 0.31	0.578
APTT (s)	33.62 ± 7.69	33.87 ± 7.38	32.49 ± 8.90	*t* = 2.79	0.095
Cr (μmol/L)	85.06 ± 68.62	84.80 ± 72.07	86.28 ± 50.10	*t* = 0.04	0.84
MON (10^9^ /L)	0.73 ± 0.77	0.74 ± 0.84	0.67 ± 0.27	*t* = 0.79	0.374
HB (g/L)	123.29 ± 20.49	123.27 ± 20.87	123.41 ± 18.71	*t* = 0.00	0.951
HCT (%)	37.03 ± 10.52	36.57 ± 7.28	39.15 ± 19.28	*t* = 5.29	**0.022**
UR (mmol/L)	6.19 ± 3.69	6.12 ± 3.52	6.48 ± 4.38	*t* = 0.80	0.371
UA (μmol/L)	310.54 ± 106.87	311.01 ± 107.69	308.34 ± 103.50	*t* = 0.06	0.815
GGT (U/L)	41.82 ± 53.95	40.83 ± 53.11	46.32 ± 57.71	*t* = 0.90	0.343
TBIL (μmol/L)	11.34 ± 9.38	11.37 ± 9.79	11.18 ± 7.22	*t* = 0.04	0.851
ALT (U/L)	23.70 ± 65.95	21.15 ± 18.97	35.42 ± 150.50	*t* = 4.10	**0.043**
AST (U/L)	25.24 ± 30.63	24.12 ± 20.86	30.41 ± 56.88	*t* = 3.69	0.055
**Hypertension**				χ^2^ = 50.13	**< 0.001**
No	349 (58.95%)	319 (65.64%)	30 (28.30%)		
Yes	243 (41.05%)	167 (34.36%)	76 (71.70%)		
**CVD**					
No	361 (60.98%)	323 (66.46%)	38 (35.85%)	χ^2^ = 34.27	**< 0.001**
Yes	231 (39.02%)	163 (33.54%)	68 (64.15%)		
**Paraplegia**				χ^2^ = 87.12	**< 0.001**
No	521 (88.01%)	456 (93.83%)	65 (61.32%)		
Yes	71 (11.99%)	30 (6.17%)	41 (38.68%)		
**Gender**				χ^2^ = 3.66	0.056
Female	137 (23.14%)	120 (24.69%)	17 (16.04%)		
Male	455 (76.86%)	366 (75.31%)	89 (83.96%)		
**DM**				χ^2^ = 1.20	0.273
No	506 (85.47%)	419 (86.21%)	87 (82.08%)		
Yes	86 (14.53%)	67 (13.79%)	19 (17.92%)		
**Hyperlipidemia**				χ^2^ = 5.79	**0.016**
No	512 (86.49%)	428 (88.07%)	84 (79.25%)		
Yes	80 (13.51%)	58 (11.93%)	22 (20.75%)		

SII, systemic immune-inflammation index; SaO_2_, arterial oxygen saturation; PT, prothrombin time; APTT, activated partial thromboplastin time; Cr, creatinine; MON, monocyte; HB, hemoglobin; ALT, alanine aminotransferase; AST, aspartate aminotransferase; CVD, cardiovascular disease; DM, diabetes mellitus. Bold values indicate that the variable is statistically significant at *P* < 0.05.

Baseline characteristics of the internal validation cohort are summarized in Table 4. A total of 212 non-CI patients and 42 CI patients were included. The mean age of the cohort was 76.51 ± 9.87 years. There were 176 males (69.29%) and 78 females (30.71%). Compared with the non-CI group, patients in the CI group tended to be older and had lower arterial oxygen saturation (SaO_2_; Table 4).

**Table 4:** Baseline characteristics of the internal validation cohort.

**Variables**	**Total (*n* = 254)**	**No cerebral infarction (*n* = 212)**	**Cerebral infarction (*n* = 42)**	**Statistic**	***p*-Value**
SII (10^9^/L)	1,429.88 ± 1,287.35	1,364.41 ± 1,186.01	1,760.38 ± 1,689.28	*t* = 3.347	0.068
Age (years)	76.51 ± 9.87	75.60 ± 9.83	81.10 ± 8.79	*t* = 11.3	**< 0.001**
SaO_2_ (%)	94.07 ± 4.13	94.65 ± 3.23	91.18 ± 6.42	*t* = 27.334	**< 0.001**
PT (s)	13.09 ± 4.01	12.99 ± 4.07	13.59 ± 3.73	*t* = 0.774	0.380
APTT (s)	32.95 ± 7.41	32.71 ± 7.30	34.16 ± 7.93	*t* = 1.351	0.246
Cr (μmol/L)	83.14 ± 51.73	80.21 ± 50.31	97.95 ± 56.74	*t* = 4.176	**0.042**
MON (10^9^ /L)	0.64 ± 0.30	0.64 ± 0.30	0.67 ± 0.32	*t* = 0.414	0.521
HB (g/L)	124.79 ± 25.03	125.61 ± 25.72	120.67 ± 20.98	*t* = 1.37	0.243
HCT (%)	37.86 ± 8.30	38.32 ± 8.44	35.52 ± 7.19	*t* = 4.032	**0.046**
UR (mmol/L)	6.15 ± 3.81	5.96 ± 3.65	7.12 ± 4.48	*t* = 3.304	0.070
UA (μmol/L)	324.75 ± 121.87	321.41 ± 122.88	341.65 ± 116.57	*t* = 0.967	0.326
GGT (U/L)	42.09 ± 47.87	42.60 ± 49.31	39.48 ± 40.29	*t* = 0.148	0.701
TBIL (μmol/L)	11.50 ± 7.40	11.62 ± 7.80	10.89 ± 4.91	*t* = 0.336	0.563
ALT (U/L)	21.37 ± 28.91	20.39 ± 19.24	26.30 ± 56.78	*t* = 1.465	0.227
AST (U/L)	26.79 ± 37.01	25.91 ± 28.87	31.21 ± 64.34	*t* = 0.718	0.398
**Hypertension**				χ^2^ = 17.288	**< 0.001**
No	157 (61.81%)	143 (67.45%)	14 (33.33%)		
Yes	97 (38.19%)	69 (32.55%)	28 (66.67%)		
**CVD**				χ^2^ = 26.302	**< 0.001**
No	166 (65.35%)	153 (72.17%)	13 (30.95%)		
Yes	88 (34.65%)	59 (27.83%)	29 (69.05%)		
**Paraplegia**				χ^2^ = 47.185	**< 0.001**
No	227 (89.37%)	202 (95.28%)	25 (59.52%)		
Yes	27 (10.63%)	10 (4.72%)	17 (40.48%)		
**Gender**				χ^2^ = 6.378	**0.012**
Female	78 (30.71%)	72 (33.96%)	6 (14.29%)		
Male	176 (69.29%)	140 (66.04%)	36 (85.71%)		
**DM**				χ^2^ = 4.469	**0.035**
No	224 (88.19%)	191 (90.09%)	33 (78.57%)		
Yes	30 (11.81%)	21 (9.91%)	9 (21.43%)		
**Hyperlipidemia**				χ^2^ = 0.878	0.349
No	233 (91.73%)	196 (92.45%)	37 (88.10%)		
Yes	21 (8.27%)	16 (7.55%)	5 (11.90%)		

SII, systemic immune-inflammation index; SaO_2_, arterial oxygen saturation; PT, prothrombin time; APTT, activated partial thromboplastin time; Cr, creatinine; MON, monocyte; HB, hemoglobin; ALT, alanine aminotransferase; AST, aspartate aminotransferase; CVD, cardiovascular disease; DM, diabetes mellitus. Bold values indicate that the variable is statistically significant at *P* < 0.05.

Baseline characteristics of the external verification cohort are summarized in Table 5. A total of 226 non-CI patients and 64 CI patients were included. The mean age of the cohort was 76.09 ± 10.15 years. There were 214 males (73.79%) and 76 females (26.21%). Compared with the non-CI group, patients in the CI group tended to be older and had higher systemic immune-inflammation index (SII) levels, along with lower arterial oxygen saturation (SaO_2_; Table 5).

**Table 5:** Baseline characteristics of the external verification cohort.

**Variables**	**Total (*n* = 290)**	**No cerebral infarction (*n* = 226)**	**Cerebral infarction (*n* = 64)**	**Statistic**	***p*-Value**
SII (10^9^/L)	1,290.98 ± 1,416.01	1,088.58 ± 775.43	2,005.73 ± 2,526.75	*t* = 22.479	**< 0.001**
Age (years)	76.09 ± 10.15	74.54 ± 10.05	81.56 ± 8.51	*t* = 25.944	**< 0.001**
SaO_2_ (%)	96.95 ± 5.95	97.47 ± 3.05	95.10 ± 11.17	*t* = 8.068	**0.005**
PT (s)	11.99 ± 2.30	11.88 ± 2.25	12.36 ± 2.45	*t* = 2.146	0.144
APTT (s)	25.07 ± 5.60	25.09 ± 5.21	24.99 ± 6.86	*t* = 0.018	0.893
Cr (μmol/L)	86.25 ± 25.96	84.54 ± 12.52	92.28 ± 49.85	*t* = 4.488	**0.035**
MON (10^9^ /L)	0.80 ± 1.70	0.84 ± 1.92	0.67 ± 0.34	*t* = 0.464	0.496
HB (g/L)	126.07 ± 20.90	128.23 ± 19.70	118.44 ± 23.31	*t* = 11.325	**< 0.001**
HCT (%)	0.69 ± 5.09	0.77 ± 5.77	0.39 ± 0.12	*t* = 0.279	0.598
UR (mmol/L)	6.27 ± 4.61	5.89 ± 4.71	7.62 ± 3.97	*t* = 7.186	**0.008**
UA (μmol/L)	331.80 ± 121.74	327.92 ± 119.29	345.49 ± 130.10	*t* = 1.038	0.309
GGT (U/L)	40.17 ± 49.80	39.49 ± 52.18	42.56 ± 40.55	*t* = 0.189	0.664
TBIL (μmol/L)	10.72 ± 8.24	10.56 ± 7.75	11.27 ± 9.83	*t* = 0.365	0.546
ALT (U/L)	20.36 ± 16.71	19.29 ± 15.96	24.15 ± 18.75	*t* = 4.269	**0.040**
AST (U/L)	27.22 ± 29.30	24.96 ± 26.98	35.17 ± 35.43	*t* = 6.16	**0.014**
**Hypertension**				χ^2^ = 68.99	**< 0.001**
No	200 (68.97%)	183 (80.97%)	17 (26.56%)		
Yes	90 (31.03%)	43 (19.03%)	47 (73.44%)		
**CVD**				χ^2^ = 19.993	**< 0.001**
No	198 (68.28%)	169 (74.78%)	29 (45.31%)		
Yes	92 (31.72%)	57 (25.22%)	35 (54.69%)		
**Paraplegia**				χ^2^ = 27.319	**< 0.001**
No	278 (95.86%)	224 (99.12%)	54 (84.38%)		
Yes	12 (4.14%)	2 (0.88%)	10 (15.62%)		
**Gender**				χ^2^ = 7.018	**0.008**
Female	76 (26.21%)	51 (22.57%)	25 (39.06%)		
Male	214 (73.79%)	175 (77.43%)	39 (60.94%)		
**DM**				χ^2^ = 2.828	0.093
No	268 (92.41%)	212 (93.81%)	56 (87.50%)		
Yes	22 (7.59%)	14 (6.19%)	8 (12.50%)		
**Hyperlipidemia**				χ^2^ = 1.73	0.188
No	248 (85.52%)	190 (84.07%)	58 (90.62%)		
Yes	42 (14.48%)	36 (15.93%)	6 (9.38%)		

SII, systemic immune-inflammation index; SaO_2_, arterial oxygen saturation; PT, prothrombin time; APTT, activated partial thromboplastin time; Cr, creatinine; MON, monocyte; HB, hemoglobin; ALT, alanine aminotransferase; AST, aspartate aminotransferase; CVD, cardiovascular disease; DM, diabetes. Bold values indicate that the variable is statistically significant at *P* < 0.05.

### 3.2 Univariate and multivariate analyses

Table 6 presents the results of both univariate and multivariate analyses in the training cohort. Variables with *p* < 0.05 from the baseline analysis of the training cohort were included in the univariate logistic regression. The univariate analysis identified a significant association between the incidence of cerebral infarction (CI) in COPD patients and the following variables: systemic immune-inflammation index (SII), age, arterial oxygen saturation (SaO_2_), hypertension, cardiovascular disease (CVD), paraplegia, and hyperlipidemia. In the multivariate logistic regression analysis, SII (*p* = 0.002; OR: 1.00), age (*p* = 0.001; OR: 1.05), SaO_2_ (*p* < 0.001; OR: 1.11), hypertension (*p* < 0.001; OR: 3.24), CVD (*p* < 0.001; OR: 2.50), and paraplegia (*p* < 0.001; OR: 5.20) were identified as independent risk factors for CI in COPD patients.

**Table 6:** Univariable and multivariable analyses of the training cohort.

**Variables**	**Univariate analysis**					**Multivariate analysis**				
	β	**S.E**	***Z***	***p*** **-Value**	**OR (95% CI)**	β	**S.E**	***Z***	***p*** **-Value**	**OR (95% CI)**
SII	0.00	0.00	4.48	**< 0.001**	1.00 (1.00–1.00)	0.00	0.00	3.05	**0.002**	1.00 (1.00–1.00)
Age	0.08	0.01	6.21	**< 0.001**	1.08 (1.06–1.11)	0.05	0.02	3.27	**0.001**	1.05 (1.02–1.08)
SaO_2_	−0.09	0.02	−4.30	**< 0.001**	0.91 (0.87–0.95)	−0.09	0.02	−3.74	**< 0.001**	0.91 (0.87–0.96)
HCT (%)	0.02	0.01	1.70	0.089	1.02 (1.00–1.05)					
ALT (U/L)	0.00	0.00	1.24	0.215	1.00 (1.00–1.01)					
**Hypertension**										
No					Reference					Reference
Yes	1.58	0.24	6.69	**< 0.001**	4.84 (3.08–7.78)	1.18	0.27	4.30	**< 0.001**	3.24 (1.91–5.60)
**CVD**										
No					Reference					Reference
Yes	1.27	0.22	5.65	**< 0.001**	3.55 (2.30–5.54)	0.92	0.26	3.49	**< 0.001**	2.50 (1.50–4.22)
**Paraplegia**										
No					Reference					Reference
Yes	2.26	0.27	8.24	**< 0.001**	9.59 (5.63–16.55)	1.65	0.31	5.24	**< 0.001**	5.20 (2.81–9.70)
**Hyperlipidemia**										
No					Reference					
Yes	0.66	0.28	2.38	**0.018**	1.93 (1.10–3.29)					

SII, systemic immune-inflammation index; SaO_2_, arterial oxygen saturation; CVD, cardiovascular disease. Bold values indicate that the variable is statistically significant at *P* < 0.05.

### 3.3 Diagnostic performance indicators of regression models

Table 7 shows the diagnostic performance indicators of the multiple logistic regression model, with a 95% CI for sensitivity of 37.7% (28.5%−47.7%), a 95% CI for specificity of 96.3% (94.2%−97.8%), a 95% CI for positive predictive value (PPV) of 69.0% (55.5%−80.5%), a 95% CI for negative predictive value (NPV) of 87.6% (84.5%−90.3%), and a 95% CI for diagnostic accuracy of 85.8% (82.7%−88.5%).Specificity, PPV, NPV and AUC were all high, and the regression model had good predictive ability.

**Table 7:** Diagnostic performance metrics of the multivariate logistic regression model.

**Metric**	**Estimate (95% CI)**	**Sample size**
Sensitivity	37.7% (28.5%−47.7%)	0.377
Specificity	96.3% (94.2%−97.8%)	0.963
Positive predictive value (PPV)	69.0% (55.5%−80.5%)	0.69
Negative predictive value (NPV)	87.6% (84.5%−90.3%)	0.876
Accuracy	85.8% (82.7%−88.5%)	0.858

All metrics were calculated on the train cohort (*n* = 592).

### 3.4 Development of the nomogram model for predicting CI in COPD patients

**Figure 1:**
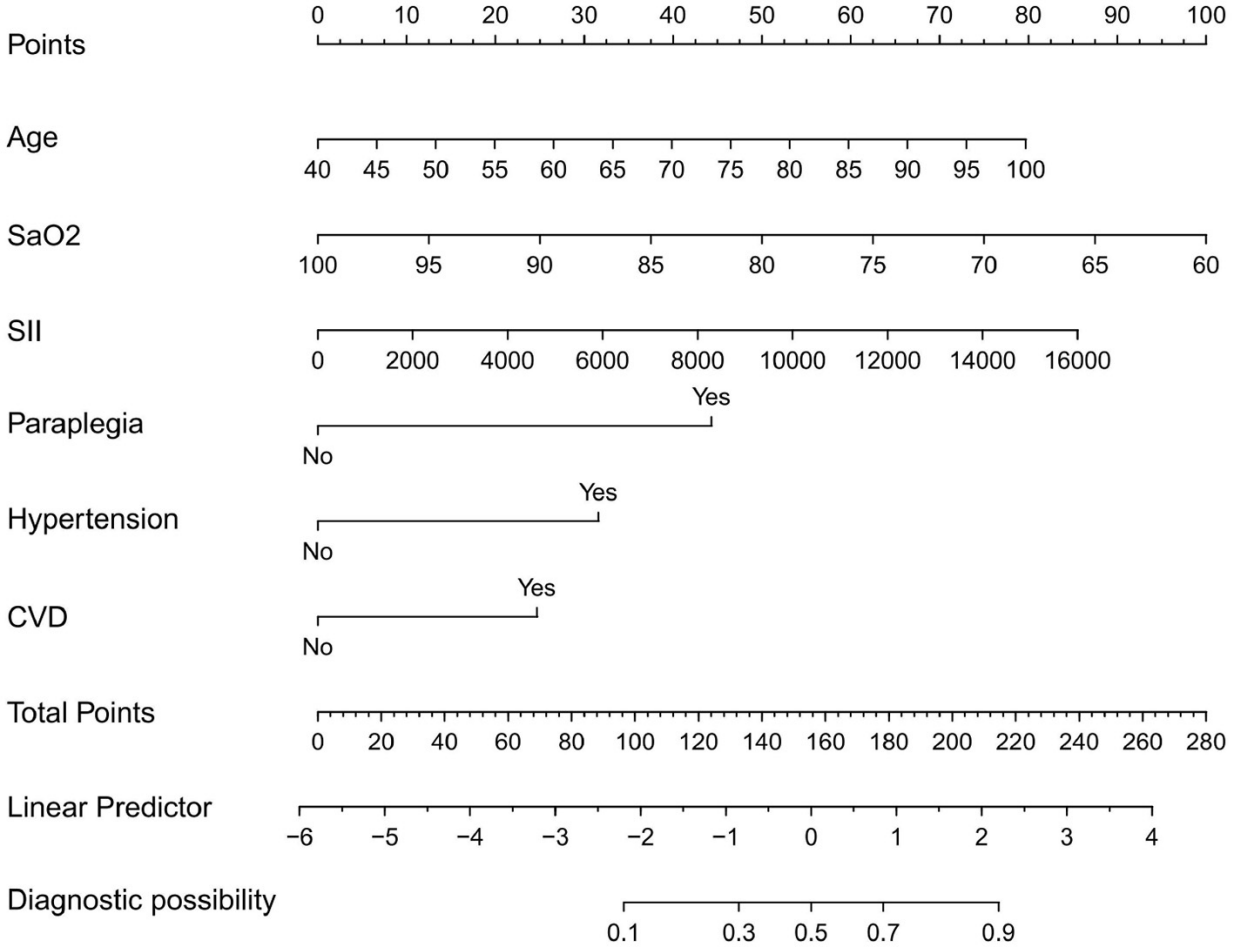
Nomogram for predicting the risk of cerebral infarction in COPD patients. SII, systemic immune-inflammation index; SaO_2_, arterial oxygen saturation; CVD, cardiovascular disease.

Based on the results of multivariate analysis, we constructed a nomogram model to predict the probability of cerebral infarction (CI) in patients with chronic obstructive pulmonary disease (COPD; Figure 1). The nomogram incorporates six predictors: systemic immune-inflammation index (SII), age, arterial oxygen saturation (SaO_2_), hypertension, cardiovascular disease (CVD), and paraplegia. Using this nomogram model, the probability of CI occurrence in individual COPD patients can be quantitatively estimated. For example, applying the model to a 70-year-old COPD patient with SaO_2_ of 80%, an SII of 14,000 × 10^9^/L, the presence of paraplegia, and no history of hypertension or cardiovascular disease, yields a total score of 209, corresponding to an estimated CI risk of 85%.

### 3.5 Validation and predictive accuracy of the nomogram model

**Figure 2:**
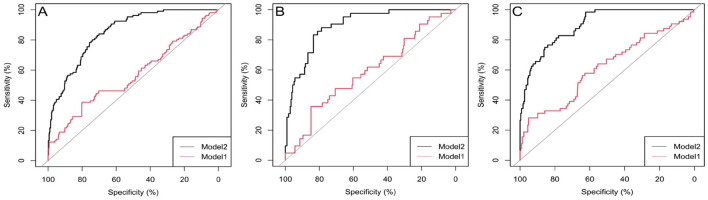
Area under the curve (AUC) of the nomogram for predicting cerebral infarction in COPD patients. **(A)** training group; **(B)** internal validation group; **(C)** external verification group. Model 1: SaO_2_; Model 2: age, SII, SaO_2_, hypertension, CVD, paraplegia. SII, systemic immune-inflammation index; SaO_2_, arterial oxygen saturation; CVD, cardiovascular disease.

**Figure 3:**
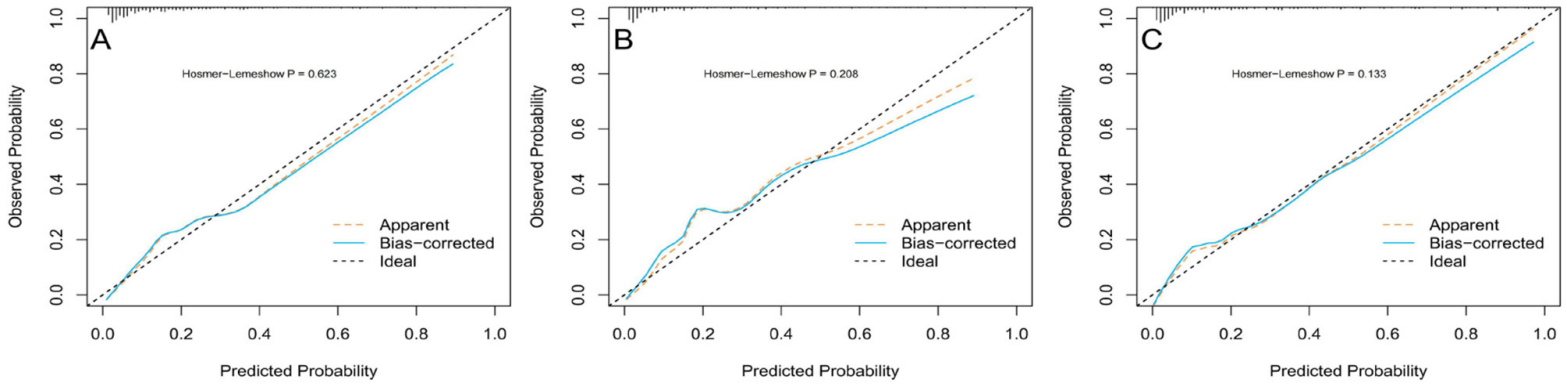
Optimized calibration curve of the nomogram for predicting the probability of cerebral infarction in COPD patients. **(A)** Training group; **(B)** internal validation group; **(C)** external verification group.

**Figure 4:**
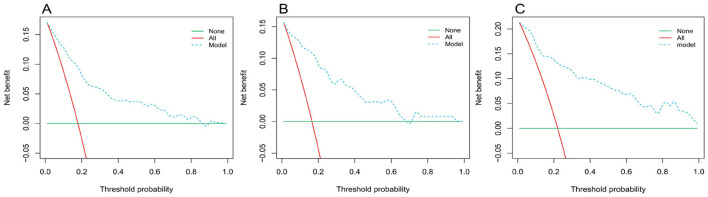
Optimized decision curve analysis of the nomogram for predicting cerebral infarction in COPD patients. **(A)** Training group; **(B)** internal validation group; **(C)** external verification group.

The initial model (Model 1) was validated with the inclusion of only one variable: arterial oxygen saturation (SaO_2_). The adjusted model (Model 2) incorporates six variables: systemic immune-inflammation index (SII), age, arterial oxygen saturation (SaO_2_), hypertension, cardiovascular disease (CVD), and paraplegia. Validation and Predictive Accuracy of the Nomogram Model the AUC (Area Under the Curve) for Model 1 in the training cohort, calculated using R software, was 0.76 (95% CI: 0.69–0.82). After adjustment, Model 2 achieved an AUC of 0.85 (95% CI: 0.82–0.89). In the internal validation cohort, Model 1 had an AUC of 0.76 (95% CI: 0.70–0.82), while the adjusted Model 2 achieved an AUC of 0.89 (95% CI: 0.85–0.94). For the external validation cohort, the AUC of Model 1 was 0.62 (95% CI: 0.53–0.70), and that of the adjusted Model 2 was 0.90 (95% CI: 0.86–0.94; Table 8). In the training cohort, the accuracy, sensitivity, and specificity of Model 1 before adjustment were 0.89 (95% CI: 0.86–0.91), 0.76 (95% CI: 0.66–0.83), and 0.92 (95% CI: 0.89–0.94), respectively. After adjustment, Model 2 demonstrated an accuracy of 0.73 (95% CI: 0.70–0.77), a sensitivity of 0.83 (95% CI: 0.75–0.80), and a specificity of 0.71 (95% CI: 0.67–0.75; Table 8). These findings suggest that SaO_2_ can reliably predict the occurrence of cerebral infarction (CI) in COPD patients. The adjusted Model 2 exhibited superior discriminative ability (Figure 2). Calibration curves demonstrated good agreement between predicted and observed outcomes in the training, internal, and external validation cohorts (Figure 3). Decision curve analysis (DCA) showed that the net benefit of the predictive model was greater across a threshold probability range of 5%−85% (Figure 4).

**Table 8:** Predictive performance analysis of the CI nomogram in COPD patients.

**Data**	**Model**	**AUC (95% CI)**	**Accuracy (95% CI)**	**Sensitivity (95% CI)**	**Specificity (95% CI)**
Train	Model 1	0.76 (0.69–0.82)	0.89 (0.86–0.91)	0.76 (0.66–0.83)	0.92 (0.89–0.94)
	Model 2	0.85 (0.82–0.89)	0.73 (0.70–0.77)	0.83 (0.75–0.80)	0.71 (0.67–0.75)
Internal validation	Model 1	0.76 (0.70–0.82)	0.89 (0.86–0.91)	0.76 (0.66–0.83)	0.92 (0.89–0.94)
	Model 2	0.89 (0.85–0.94)	0.80 (0.75–0.85)	0.88 (0.75–0.95)	0.79 (0.73–0.84)
External validation	Model 1	0.62 (0.53–0.70)	0.80 (0.75–0.84)	0.23 (0.19–0.40)	0.95 (0.92–0.97)
	Model 2	0.90 (0.86–0.94)	0.83 (0.79–0.87)	0.75 (0.63–0.84)	0.86 (0.81–0.90)

Model 1: SaO_2_; Model 2: age, SII, SaO_2_, hypertension, CVD, paraplegia. SII, systemic immune-inflammation index; SaO_2_, arterial oxygen saturation; CVD, cardiovascular disease.

## 4 Discussion

This study is the first to develop and validate a nomogram prediction model for the risk of CI in patients with COPD. The majority of previous studies investigated the correlation between a single factor, such as age, hypoxemia, and hypertension, and the occurrence of CI in COPD patients in isolation, lacking a comprehensive evaluation system integrates multi-dimensional indicators. Also, the results of traditional statistical models, such as logistic regression, are usually presented as odds ratios (OR) or hazard ratios (HR), can explain the association of factors but cannot provide a visual and convenient tool for clinicians to quantify the risk of disease in individual patients. Therefore, we retrospectively analyzed the clinical data of 846 COPD patients at Guangdong Medical University Affiliated Hospital from June 2018 to December 2019, with 592 in the training group and 254 in the internal validation group. We also prospectively collected clinical data of 290 COPD patients at Guangdong Medical University Affiliated Second Hospital from January 2024 to October 2024 for external validation. It was observed that CI patients have characteristics such as older age, higher levels of hematocrit, alanine aminotransferase, SII, and lower levels of SaO_2_. Logistic regression analysis showed *p* < 0.05 for factors such as SII, Age, SaO_2_, Hypertension, CVD, and Paraplegia, which are considered independent risk factors for CI in COPD patients. We established a nomogram model for predicting the probability of ICU admission in patients with COPD based on SaO_2_, and the modified model included six variables: systemic immune-inflammation index (SII), age, arterial oxygen saturation (SaO_2_), hypertension, cardiovascular disease (CVD) and paraplegia, which was used to predict the probability of critical illness in patients with COPD. The AUC 95% CI was 0.85 (082–0.89) for the training set; the AUC 95% CI was 0.89 (0.85–0.9) for the internal validation set; the AUC 95% CI was 0.90 (0.86–0.94) for the external validation, demonstrating good predictive performance and calibration of the nomogram model.

Arterial oxygen saturation (SaO_2_) reflects the oxygen-carrying capacity of hemoglobin in the blood and is expressed as a percentage. It serves as an essential biological indicator for assessing the body's adaptation to hypoxia. Therefore, SaO_2_ is considered a major clinical indicator for evaluating hypoxia in COPD patients. Previous studies have found that low arterial oxygen tension can significantly aggravate brain injury caused by cerebral hypoperfusion. Lower SaO_2_ levels are associated with more severe periventricular white matter lesions (WML), and WMLs tend to be more pronounced in COPD patients, indicating that hypoxia caused by low SaO_2_ may contribute to more severe cerebral damage (18). Age has also been linked to CI, with imaging-confirmed stroke patients being significantly older than those without stroke (19). Hypertension is strongly associated with stroke, with studies confirming its role in vascular injury and thromboembolic events (20). Kannel's 18-year follow-up study in Framingham and Massachusetts found that individuals with blood pressure above 160/95 mmHg had a sevenfold increased risk of stroke compared to normotensive individuals (21). Moreover, studies by Prineas and Marshall (22) demonstrated that antihypertensive treatment could significantly reduce the incidence of all stroke types. Numerous studies have confirmed correlations between immune-inflammatory cell ratios in routine blood tests—such as the neutrophil-to-lymphocyte ratio and platelet-to-lymphocyte ratio—and acute ischemic stroke (23). Inflammatory cells and mediators contribute to the formation, progression, and rupture of atherosclerotic plaques, which are key pathological mechanisms of ischemic stroke (24). Therefore, the role of SII in the development of CI is biologically plausible.

Various cardiovascular diseases also influence CI risk. For example, (1) atrial fibrillation is associated with 20%−30% of all ischemic strokes and 10% of cryptogenic strokes, primarily due to thrombus formation in the left atrial appendage (LAA) (25); (2) large myocardial infarctions (MI) may lead to cardiovascular failure, potentially reducing cerebral perfusion and causing watershed infarcts or diffuse hypoxic-ischemic brain injury (26).

However, the pathophysiological mechanisms of stroke are complex, and stroke classification has been carried out based on the different pathophysiological mechanisms of stroke (27). Atherosclerosis and cardiogenic embolism are the two most common causes of ischemic stroke. Major atherosclerosis is related to factors such as aortic plaque and metabolic syndrome. These factors may be related to coronary atherosclerosis. The 2-year recurrence rate of ischemic events is high, while the incidence rate of cardiovascular events is 18.6%, and the vascular mortality rate is 8.8% (28). The incidence of different sources of cardioembolic events related to cerebral ischemic events and the inconsistent prognosis of cardioembolic stroke caused by these diseases (29). The occurrence of large artery occlusion caused by cardioembolic events is more rapid, the establishment of collateral circulation is relatively insufficient, ischemic tolerance is relatively poor, the treatment time window is short, and the risk of bleeding transformation after vascular opening is high (30). Therefore, the prognosis of the two types of ischemic stroke mentioned above is poor. However, lacunar ischemic stroke is caused by small infarctions in the unilateral perforating blood supply area, and according to different research data, this type of stroke accounts for ~11%−27% of acute strokes. This disease is not only a common type of stroke, but also the first clinical manifestation of cerebral small vessel disease, which may lead to serious physical dysfunction and vascular cognitive impairment in the long term (31). The prognosis and mechanisms of each type of ischemic stroke vary, and we need to pay attention to the risk factors for different types of stroke in COPD patients, as there may be differences.

This study also has certain shortcomings. Firstly, as it is a retrospective study, there may be some information bias in data collection and analysis. Secondly, there are differences in the risk of different subtypes of ischemic stroke, but the sample size of patients with each subtype of ischemic stroke included in this study is relatively small, therefore, risk analysis of patients with each subtype of ischemic stroke cannot be conducted. Third, although we tried to include a variety of clinical variables, some unmeasured confounding factors may have affected the model. Therefore, in future studies, the sample size of patients will be increased to conduct risk analysis of various subtypes of ischemic stroke, especially to specifically evaluate the risk of ischemic stroke in COPD patients without or with myocardial infarction. And in the future, we need multicenter prospective studies to confirm the effectiveness and practicality of the line chart, as well as to conduct research on the underlying mechanisms of various risk factors.

## 5 Conclusion

COPD is prone to cerebral infarction, and we verified the relationship between COPD and cerebral infarction through a visualogram model. The incidence of cerebral infarction in COPD patients is affected by systemic immune-inflammatory index (SII), age, hypertension, cardiovascular disease (C), hemiplegia, and SaO_2_, and the nomogram model for risk prediction based on SaO_2_ has good predictive efficacy, which can provide a reference forbral infarction in COPD patients in clinical practice. However, this study was retrospective, the sample size of the different subtypes of ischemic stroke was small, the risk analysis of each subtype of ischemic stroke could not be performed, and there may be some unmeasured confounding factors, which had a certain impact on the of the study. In the future, we need multi-center prospective studies to verify the effectiveness and practicality of the nomogram, and the basic mechanism of each risk factor also to be studied.

## Data Availability

The data used to support the findings of this study are available from the corresponding author upon request.
